# Case study of colorectal endometriosis treated with endoscopic submucosal excavation

**DOI:** 10.3389/fphys.2023.1073241

**Published:** 2023-05-05

**Authors:** Tangzhou Xu, Bingfeng Qiu, Qi Xu, Junhan Qu, Dandan Zhuang, Qiancheng Xu

**Affiliations:** Department of Gastroenterology, Zhoushan Hospital, Wenzhou Medical University, Zhoushan, China

**Keywords:** endometriosis, colorectal endometriosis, endoscopic submucosal excavation, surgical treatment, CEM

## Abstract

Colorectal endometriosis (CEM) is a rare and complicated form of deep invasive endometriosis. Its treatment methods include drug therapy and surgery. However, it is often difficult to alleviate symptoms and address problems, such as infertility, using drug treatment alone. Surgical intervention provides a histologic diagnosis, allows assessment of pelvic cysts or masses with features concerning for malignancy, and reduces pain by destroying the endometriotic implants. We consider surgery in women with the following: Persistent pain despite medical therapy; Contraindications to or refusal of medical therapy; Need for a tissue diagnosis of endometriosis; Exclusion of malignancy in an adnexal mass; Obstruction of the bowel or urinary tract. But there is no consensus about the surgical methods. With the rapid development of gastroenteroscopy technology in recent years, many local gastrointestinal tumors that previously required surgical resection can now be removed by endoscopic surgery. Herein, we report one case of CEM treated by endoscopic submucosal excavation (ESE) to provide a new treatment option for the radical resection of single CEM.

## Introduction

Endometriosis (EMT) refers to the presence of endometrial tissue outside the endometrium and often occurs within parts of the reproductive system, including the ovaries, rectouterine pouch, or pouch of Douglas, and uterosacral ligaments. It can also occur in the abdominal cavity, chest cavity, and limbs (Ding et al., 2017). Colorectal endometriosis (CEM) accounts for about 3%–37% of EMT cases, with the most common site being the junction of the rectum and sigmoid colon (Roman et al., 2016). Ultrasound is the preferred imaging modality for women suspected of having rectovaginal endometriosis. Additional imaging techniques such as magnetic resonance imaging (MRI) or computed tomography (CT) can be useful for women suspected of having bowel disease proximal to the rectosigmoid colon. Although not diagnostic, imaging can identify findings highly suggestive of endometriosis and map the location and extent of disease, which is extremely important for surgical planning. However, imaging of proximal bowel is less likely to be conclusive, and disease at this level may only be visualized at the time of laparoscopy. Traditionally, the disease is treated mainly with drug therapy and surgery, with the latter being the main treatment modality due to the difficulty of curing the disease with drug therapy alone. At present, the most commonly used surgical methods in clinical practice include the resection of bowel surface lesions (rectal shaving) (Alabiso et al., 2015), the discoid resection (Roman et al., 2018), and segmental bowel resection, but there is no uniform surgical standard (Daraï et al., 2010). Recently, one case of CEM was treated with endoscopic submucosal excision (ESE) in our hospital, and an excellent curative effect was achieved. The details are reported below.

### Clinical data

A 47-year-old female patient was admitted because of “repeated abdominal pain for 5 years and a submucosal protrusion found in the rectum for 3 months” and she had experienced persistent dysmenorrhea which would alleviate spontaneously. On 19 July 2021, the patient underwent a colonoscopy in another hospital, and a submucosal ridge with a smooth surface was found 10 cm above the anus. Endoscopic ultrasonography (EUS) results from the other hospital confirmed that it originated from a hypoechoic light mass of the muscularis propria. Differential diagnoses included rectal protrusion, neuroendocrine tumor (NET), and myoma. Rectal magnetic resonance imaging (MRI) was performed, no obvious abnormality was found in the rectum, and laparoscopic treatment or outpatient follow-up was recommended.

**FIGURE 1:**
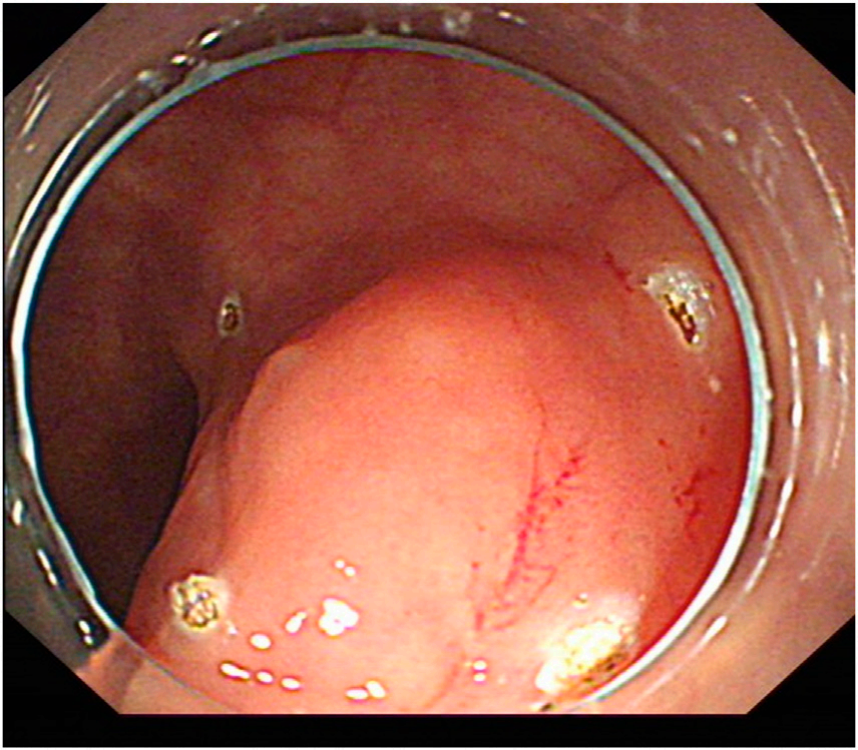
Submucosal ridge.

**FIGURE 2:**
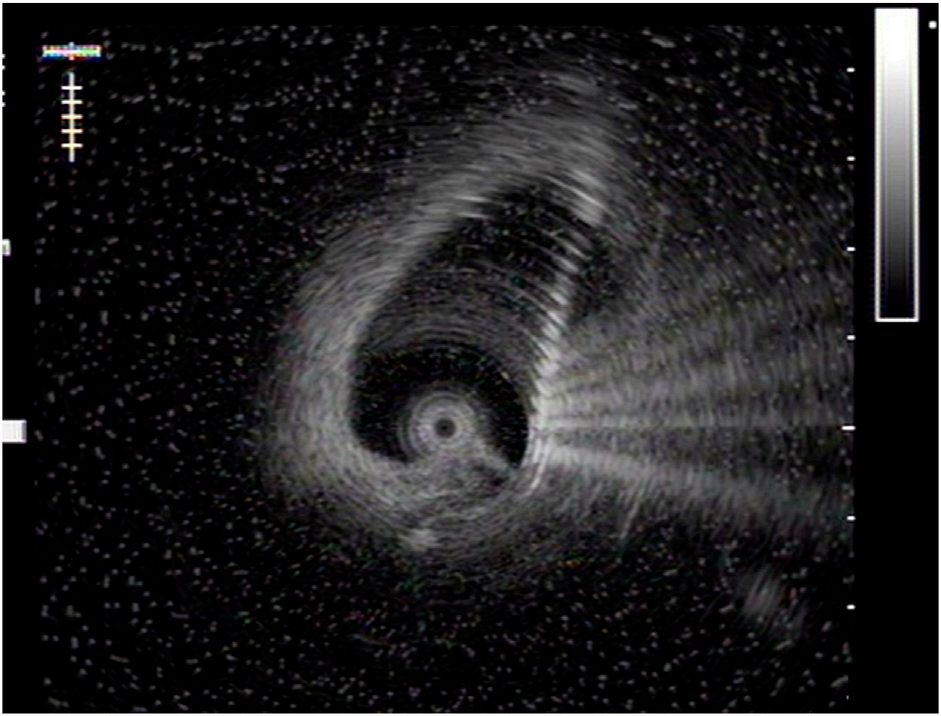
Ultrasonic image of ridge.

The patient was referred to our hospital due to her tomophobia. No obvious abnormality was found in the patient’s physical examinations, routine blood test and blood biochemistry results, and serial examinations of tumor biochemical markers at admission. A EUS reexamination revealed that a submucosal mass emerged from the third and fourth layers of intestinal tissue with a size of 13.1 × 8.2 mm and was found at 10 cm above the anus, which was a low echo cluster but with a high echo inside. (Figures 1, 2). A rectal submucosal tumor was considered, e.g., NET, stromal tumor, leiomyoma, and others.

**FIGURE 3:**
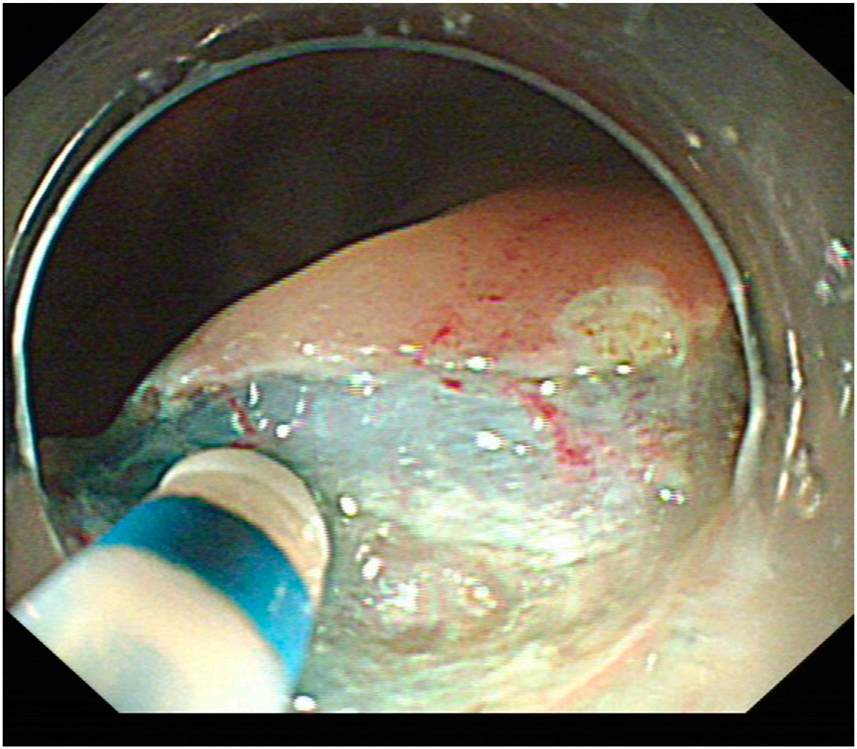
Incision after submucosal injection.

**FIGURE 4:**
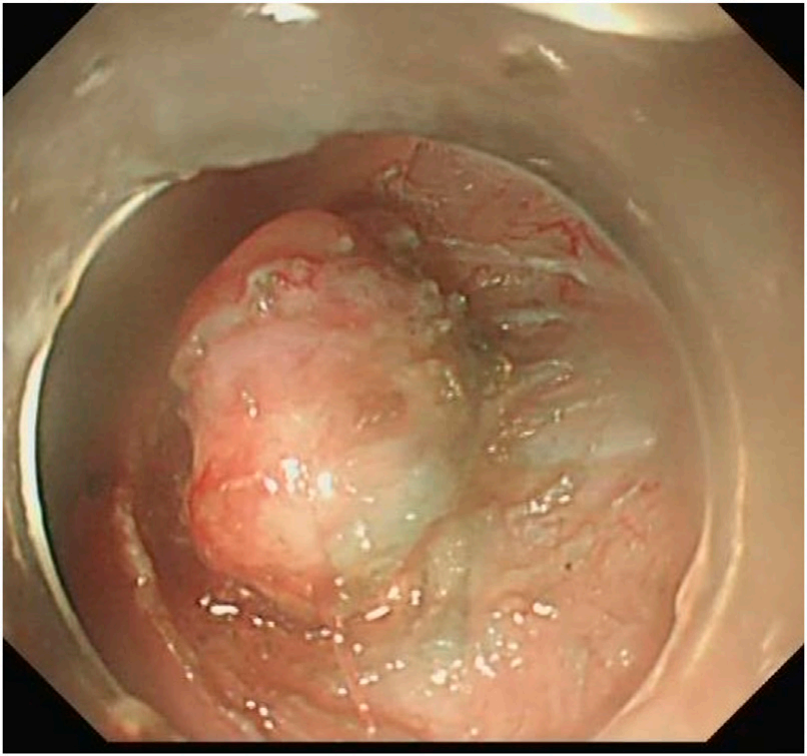
Lesion dissection.

**FIGURE 5:**
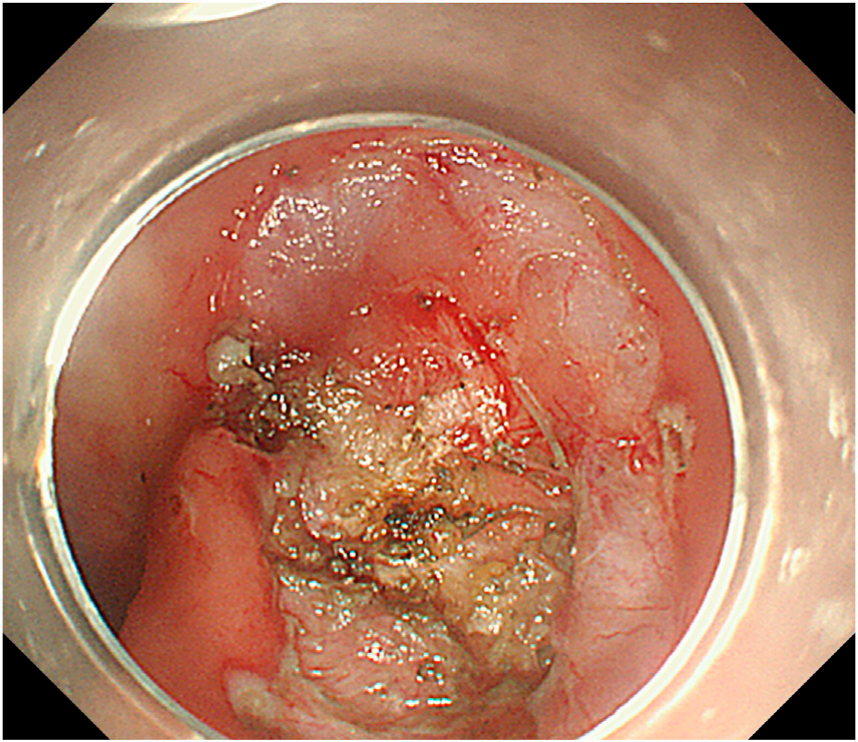
Complete dissection of lesion.

**FIGURE 6:**
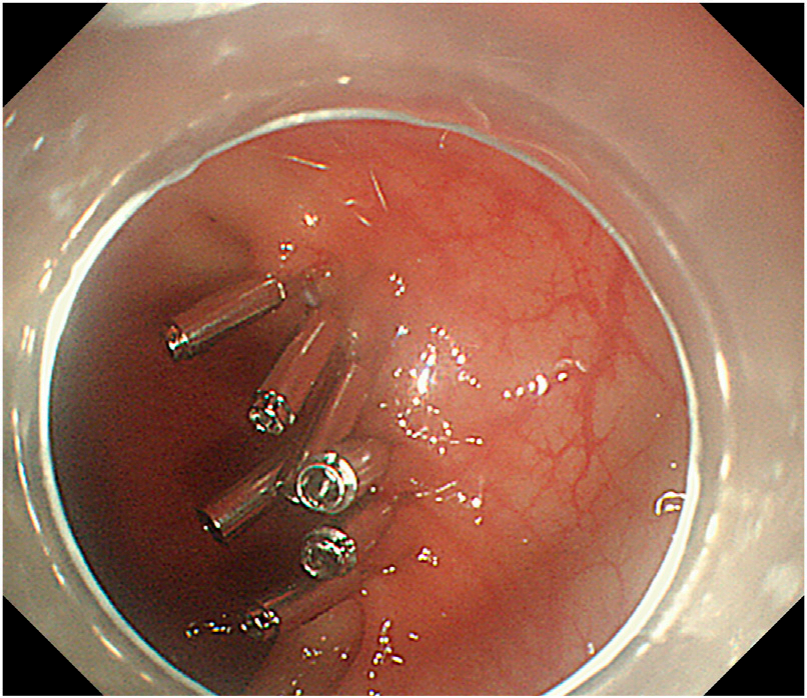
After wound tissue clipping.

**FIGURE 7:**
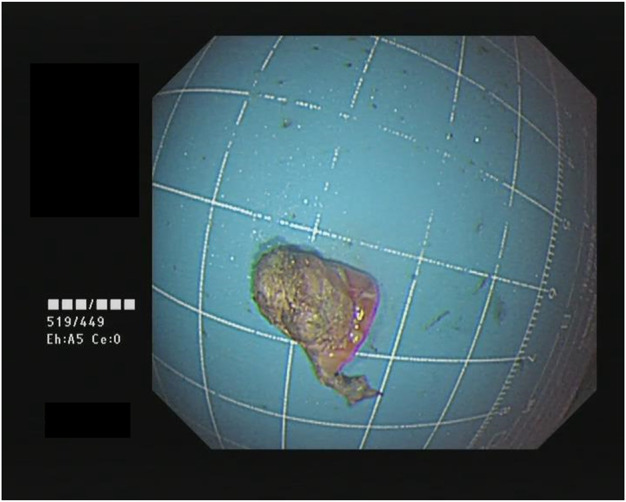
Lesion.

After obtaining informed consent from the patient, ESE under general anesthesia with tracheal intubation was performed. Markers were made around the lesion under an endoscope. After the submucosal injection of a mixture of normal saline and methylene blue, a yellowish–white mass was observed upon cutting the mucosa (Figure 3). The mass was gradually and completely stripped with a DualKnife (Figures 4, 5), and the wound was sutured with tissue clips (Figure 6). The size of the removed mass was 9 × 15 mm (Figure 7). The patient fasted for 3 days after the surgery with anti-infection and nutritional support treatment and was discharged 5 days later.

**FIGURE 8:**
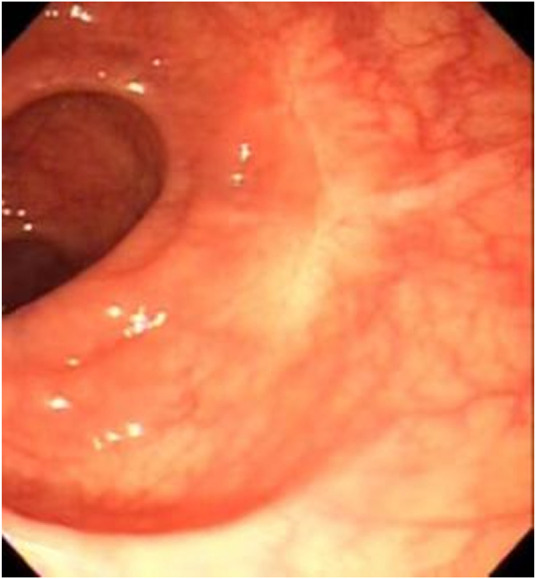
Wound re-examination image.

One week after the surgery, the patient’s pathological and immunohistochemical results were consistent with an endometrial nodule. The immunohistochemical results were as follows: CD117 (−), CD34 (−), SMA (−), Ki67 (+) 10%, Dog-1 (−), CK7 (+), CD10 (+) interstitial cells, ER (+), and PR (+). After the diagnosis of CEM, the patient was referred to the Department of Gynaecology for further hormonal therapy and was reevaluated for some other non-infiltrating external nodules on the sigmoid and rectum which may need further open or laparoscopic surgery, but without significant findings. In the postoperative follow-up, the patient’s abdominal pain had improved. Two months later, the results of an abdominal and pelvic computed tomography (CT) enhanced scan and colonoscopy performed in another hospital confirmed that the wound had healed (Figure 8), with no recurrence of the lesion.

## Discussion

Endometriosis is a disease that affects the health of fertile women. Some patients with CEM may be asymptomatic. In addition to gynecological symptoms, such as dysmenorrhea, pain during sexual intercourse, and infertility, some patients have combined gastrointestinal symptoms, such as changes in bowel habits, abdominal pain, and rectal bleeding (Bendifallah et al., 2021). To date, there are no clear evaluation guidelines for patients with bowel involvement of EMT. A review of the relevant literature revealed that for fertile women, the possibility of this disease should be considered in the following situations: (1) Patients with EMT with bowel symptoms. (2) Periodic hematochezia. (3) Incomplete bowel obstruction. (4) A bowel mass located outside the bowel mucosa or one that shrinks after menstruation. (5) A submucosal mass confirmed by colonoscopy (Régenet et al., 2001; Preoperative rectosigmoid, 2023). For patients with the above manifestations, it is recommended to undergo EUS to detect the level and scope of lesion invasion and bowel wall involvement via colonoscopy in combination with MRI and other imaging examinations to improve the diagnostic rate and guide subsequent treatment. Several studies have confirmed that EUS is effective for diagnosing bowel EMT and evaluating the involved and infiltrated bowel wall (Ferrari et al., 2012; Working group of ESGE ESHRE and WES Saridogan et al., 2017).

Surgical intervention provides a histologic diagnosis, allows assessment of masses with features concerning for malignancy, especially for those who have persistent pain despite medical therapy, with contraindications to or refusal of medical therapy, need for a tissue diagnosis of endometriosis, and with obstruction of the bowel or urinary tract. Though there is no uniform surgical standard (ACOG Committee Opinion No, 2023; Diagnosis and management, 2023; Necessary For Deep Endometriosis, 2023), according to the relevant literature, there are two main kinds of traditional surgical treatment for CEM. The first is the resection of the pathogenic bowel segment; however, this is relatively traumatic, with complications such as bowel obstruction and nerve injury. The second is rectal shaving or resection of ectopic lesions and the surrounding normal bowel wall tissues, including full-thickness rectal resection and discoid resection, which is relatively minimally invasive, and the length and function of the intestine can be preserved after surgery (Diana et al., 2011; Tan-Kim et al., 2015). The treatment goals of clinicians in recent years have been to improve the clinical symptoms of patients to the maximum extent, improve the fertility of women of childbearing age, and minimize the risks and complications related to surgery (Régenet et al., 2001; Grigoriadis et al., 2022). The advent of totally laparoscopic intracorporeal anastomosis with natural orifice specimen extraction (NOSE) brings the concept of minimally invasive surgery into full play. It is reported that the natural lumens of the human body can be used for the relevant surgical operations of CEM treatment via the anus and vagina, including laparoscopic transvaginal lesion resection + transvaginal specimen extraction and laparoscopic transanal lesion resection + transvaginal specimen extraction; this can not only avoid the need for additional incisions but also reduce both surgical trauma and the incidence of postoperative complications (Bokor et al., 2018; Qi et al., 2019). However, although NOSE has good efficacy, its popularization is difficult.

Endoscopic resection technology has made rapid progress in recent years, enabling many local gastrointestinal tumors that previously required surgical resection to be removed by endoscopic surgery. Gastrointestinal submucosal tumors cannot be accurately diagnosed via routine mucosal biopsy due to the depth of their lesions, and they rely mainly on empirical diagnosis under EUS, which requires the pathological testing of specimens after resection. The main indications of the ESE technique are benign or low-grade submucosal tumors (such as stromal tumors, carcinoids, and leiomyomas) with a length of no more than 3 cm. According to the shape and growth position of the tumor, the surgical method can be direct excavation, submucosal tunneling resection, or even full-layer resection of the digestive tract wall, with surgical incisions sutured under an endoscope (Endoscopic resection, 2023).

Before surgery, the case in this study was considered to be a rectal submucosal tumor, which corresponded with the indication of ESE. However, after the surgery, the pathological diagnosis was an EMT nodule, indicating the feasibility of using endoscopic resection for small rectal EMT lesions. Although endoscopic technology is advancing rapidly, its technical requirements and difficulties are relatively high. Therefore it must be performed by an experienced endoscopic physician. Furthermore, the surgery must be performed in an advanced medical diagnosis and treatment center because of the potential for complications, such as perforation and bleeding (Milone et al., 2015).

In this case, the CEM was resected with ESE with little trauma, rapid recovery, and a satisfactory short-term effect. It provides a new treatment method for the radical resection of small single CEM. However, the long-term therapeutic effect still requires further investigation in a wider range of clinical trials at a higher level.

## Data Availability

The original contributions presented in the study are included in the article/supplementary material further inquiries can be directed to the corresponding author.

## Abbreviations

EMT, endometriosis; CEM, Colorectal endometriosis; ESE, endoscopic submucosal excision; MRI, magnetic resonance imaging; NOSE, totally laparoscopic intracorporeal anastomosis with natural orifice specimen ex-traction.
